# Making a Short Story Long: Regulation of P-TEFb and HIV-1 Transcriptional Elongation in CD4^+^ T Lymphocytes and Macrophages

**DOI:** 10.3390/biology1010094

**Published:** 2012-06-15

**Authors:** Rajesh Ramakrishnan, Karen Chiang, Hongbing Liu, Sona Budhiraja, Hart Donahue, Andrew P. Rice

**Affiliations:** 1 Department of Molecular Virology & Microbiology, Baylor College of Medicine, Houston, TX 77030, USA; Email: ramakris@bcm.edu (R.R.); chiang@bcm.edu (K.C.); hliu@bcm.edu (H.L.); budhiraj@bcm.edu (S.B.); hartdonahue@gmail.com (H.D.); 2 Interdepartmental Program in Translational Biology and Molecular Medicine, Baylor College of Medicine, Houston, TX 77030, USA

**Keywords:** P-TEFb, HIV-1, Tat, T-loop phosphorylation, Cyclin T1, miRNA

## Abstract

Productive transcription of the integrated HIV-1 provirus is restricted by cellular factors that inhibit RNA polymerase II elongation. The viral Tat protein overcomes this by recruiting a general elongation factor, P-TEFb, to the TAR RNA element that forms at the 5’ end of nascent viral transcripts. P-TEFb exists in multiple complexes in cells, and its core consists of a kinase, Cdk9, and a regulatory subunit, either Cyclin T1 or Cyclin T2. Tat binds directly to Cyclin T1 and thereby targets the Cyclin T1/P-TEFb complex that phosphorylates the CTD of RNA polymerase II and the negative factors that inhibit elongation, resulting in efficient transcriptional elongation. P-TEFb is tightly regulated in cells infected by HIV-1—CD4^+^ T lymphocytes and monocytes/macrophages. A number of mechanisms have been identified that inhibit P-TEFb in resting CD4^+^ T lymphocytes and monocytes, including miRNAs that repress Cyclin T1 protein expression and dephosphorylation of residue Thr186 in the Cdk9 T-loop. These repressive mechanisms are overcome upon T cell activation and macrophage differentiation when the permissivity for HIV-1 replication is greatly increased. This review will summarize what is currently known about mechanisms that regulate P-TEFb and how this regulation impacts HIV-1 replication and latency.

## 1. Introduction

While the human genome encodes ~30,000 genes, only ~10,000–20,000 of these genes are expressed in an individual cell at any given time [1]. The regulation of gene expression therefore includes transcribed genes as well as those that are kept silent, with both classes contributing to proper cell function. The conventional view has been that gene expression is primarily regulated at the level of transcriptional initiation at the promoter, by virtue of its epigenetic makeup and the recruitment of RNA polymerase II (RNAP II) and other general transcription factors to this region. It was believed that once promoter clearance was achieved, the subsequent generation of a complete mRNA transcript, including elongation and processing, proceeded in a straightforward manner. However, recent studies [2,3,4] have shown that mRNA transcription is far more complex than this simplified view would suggest. It now appears that in addition to regulation at the promoter, a widespread rate-limiting step in gene expression occurs when the RNAP II complex pauses just after clearing the promoter. This promoter-proximal pausing is overcome with the general elongation factor P-TEFb (Positive transcription elongation factor-b), which promotes the transition into productive transcriptional elongation. P-TEFb is also an essential HIV-1 co-factor, as productive transcription of the HIV-1 provirus depends on the recruitment of P-TEFb. Owing to its importance in gene expression, the expression and availability of P-TEFb is under stringent control, and this review will focus on the various mechanisms by which P-TEFb is regulated in activated CD4^+^ T lymphocytes and differentiated macrophages, the two major cell types that support productive HIV-1 infection, and in resting CD4^+^ T lymphocytes, one of the main HIV-1 latency reservoirs. 

## 2. Overview of Transcriptional Elongation

Transcription commences with the assembly of the preinitiation complex (PIC), consisting of RNAP II and multiple general transcription factors (GTFs), on the promoter [5], followed by local melting of the promoter DNA near the transcription start site. RNA synthesis by RNAP II may initially be quite inefficient, resulting in aborted transcripts only a few nucleotides in length, but further elongation is eventually orchestrated by GTFs and the increased stability of the RNAP II complex following conformational changes induced by the nascent RNA. The GTF TFIIH then drives promoter clearance by phosphorylating residues in the C-terminal domain (CTD) of RNAP II’s largest subunit [6,7]. In mammals the CTD is comprised of 52 repeats of a consensus (Y_1_S_2_P_3_T_4_S_5_P_6_S_7_) sequence, and during the different stages of transcription, it serves as a molecular scaffold for assembly of the appropriate complexes, which is largely determined by the phosphorylation status of the serines in its heptad repeats. While RNAP II is initially recruited in the unphosphorylated state, Ser5 phosphorylation by the Cdk7 catalytic subunit of TFIIH promotes binding of GTFs involved in promoter clearance and dissociation from factors involved in transcript initiation; Ser7 is also subject to phosphorylation at this time, but the functional significance of this is still being elucidated [6,8]. 

Early elongation occurs very inefficiently, frequently resulting in the promoter-proximal pausing of RNAP II, which is also mediated by the association of two negative elongation factors, NELF and DSIF. Paused polymerases are found on both silenced and active genes, and it is increasingly clear that this is a common means of gene regulation [4,9,10], as pausing can lead to either productive elongation or termination, and pause duration may determine transcriptional output [11]. The pause may also serve to coordinate processing events, as capping of the nascent transcript occurs during this time [12]. Release of the stalled RNAP II and efficient transcriptional elongation depends on the hyperphosphorylation of the RNAP II CTD by the kinase action of core P-TEFb. In this review, we define the “core” P-TEFb as comprising Cdk9 and either Cyclin T1 (which Tat recruits) or Cyclin T2a/T2b. P-TEFb phosphorylates the Ser2 residues of the CTD, as well as the Spt5 subunit of DSIF and the RD subunit of NELF [13,14,15]. This phosphorylation displaces NELF [16] from the RNAP II and it morphs DSIF into a positive elongation factor which associates with RNAP II through the enzymes’ transit to the end of the coding sequence [13,14]. Thus, P-TEFb effectively transitions the polymerase into a processive enzyme [17]. While HIV-1 Tat recruits P-TEFb to the viral LTR, and the bromodomain protein Brd4 directs P-TEFb to cellular promoters [18], it is likely that this occurs within the context of much larger complexes, as P-TEFb was recently found to associate with the super elongation complex (SEC), a multi-protein assembly which also promotes effective transcript elongation [19]. P-TEFb also associates with c-Ski-interacting protein (SKIP), which enhances HIV-1 Tat mediated viral transcriptional elongation [20]. Using pulse-labeling and other techniques, the rate at which transcriptional elongation occurs has been reported to be between 1 and 6 kb/min [21]. A recent imaging study using fluorescent RNA tagging to track transcripts derived from individual cells containing a single HIV-1 vector integration presented data that the rate is much faster, clocking in at 50–100 kb/min [22]. 

In addition to mRNA capping, processes like splicing and RNA export are also linked to transcript elongation [18,23]. As mentioned earlier, the 5’ end of nascent transcripts are capped by the action of capping enzymes during RNAP II pausing [24], and it has been shown that the Spt5 subunit of DSIF stimulates this process [25,26]. Splicing of the elongating transcript is facilitated by the interaction of SF2/ASF and SC35 with serine 2 phosphorylated CTD [27,28,29], and increased exonic occupancy of P-TEFb can promote alternative splicing [30]. 

## 3. A Perspective on P-TEFb

RNase protection experiments in the late 1980s in the Peterlin laboratory showed that the viral transactivator protein Tat increased the processivity of RNA polymerase II [31], in a manner dependent on a section of HIV-1 RNA immediately downstream of the LTR, called the transactivation response element (TAR). Upon its transcription, the TAR RNA folds into a stem-loop structure also containing a smaller tri-nucleotide bulge which is bound by Tat. Our laboratory provided the first evidence that a kinase complex that we named TAK (Tat-associated kinase) binds specifically to HIV-1 Tat [32,33]. Around the same time an RNAP II elongation factor was isolated from *Drosophila* nuclear extracts [34] which had the ability to phosphorylate the CTD of RNAP II. This elongation factor was named positive transcription elongation factor b (P-TEFb). Intriguingly, both TAK and P-TEFb contained a 42-kDa catalytic subunit [32,33,35] subsequently shown to be closely related [36]. Following purification of P-TEFb by the Price laboratory, it was found that the 42 kDa subunit was the Drosophila orthologue of the human protein PITALRE [37]. This catalytic subunit was later renamed Cdk9 (Cyclin-dependent kinase 9) [37,38]. Cyclin-dependent kinases are regulated by an associated cyclin, and in the case of Cdk9, a novel cyclin partner called Cyclin T was found to play this regulatory role [39]. There are two Cyclin T genes in mammals, namely, Cyclin T1 and Cyclin T2, both of which were found to bind Cdk9, whereas only Cyclin T1 additionally binds to Tat and the loop region of the TAR RNA. A critical Cysteine 261 (Cys 261) residue in Cyclin T1 is conserved and is necessary for Tat mediated transactivation [40]. However, in murine cells, a tyrosine at the same position hinders the binding of Cyclin T1 to Tat [40], while a substitution to cysteine renders cells permissive for HIV-1 transcription [40,41,42]. Interestingly, equine and canine Cyclin T1 conserve the Cys261 residue, allowing Tat-mediated transactivation in these cells [43,44]. Therefore, Cyclin T1-containing P-TEFb is the key mediator of HIV-1 Tat function, and is essential for productive proviral transcription.

## 4. Expression and Regulation of P-TEFb in CD4^+^ T Cells and Macrophages Affects HIV-1 Replication

**Figure 1:**
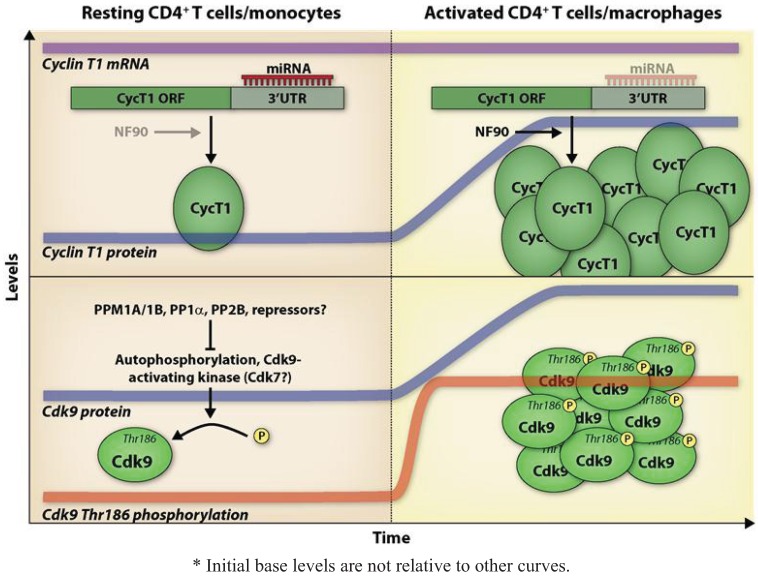
Proposed model for P-TEFb regulation in resting CD4+ T cells/monocytes *. In resting CD4^+^ T cells and monocytes, Cyclin T1 protein levels are low, with protein translation being repressed via the action of Cyclin T1-targeting miRNAs. Additionally, it has been shown that the RNA-binding protein NF90, which acts to stimulate Cyclin T1 translation, is expressed at low levels in promonocytic cells. Following CD4^+^ T cell activation or monocyte differentiation, Cyclin T1 protein levels undergo a dramatic increase independently of any increase in Cyclin T1 mRNA levels; expression of Cyclin T1-targeting miRNAs is concomitantly decreased. Activation of promonocytic cells induces NF90 expression, which further contributes to increased Cyclin T1 translation. Cell activation also leads to Cdk9 protein induction and an increase in Cdk9 Thr186 phosphorylation, which occurs very rapidly in CD4^+^ T cells (depicted here), and at a more delayed rate during macrophage differentiation. This phosphorylation is thought to be mediated by autophosphorylation or a Cdk9-activating kinase. It is unclear how low levels of Thr186-phosphorylated Cdk9 are maintained in the resting cell, although it has been shown that Thr186 dephosphorylation can be mediated by PPM1A, PPM1B, PP1α, and PP2B and it is likely that these phosphatases or other repressors are involved.

HIV-1 infection and replication is productive in activated CD4^+^ T cells and differentiated macrophages. Resting CD4^+^ T cells and monocytes can be infected, although this process is very inefficient, and these cells are refractory for HIV-1 replication. A great number of studies have been carried out to determine the blocks to HIV-1 replication in resting CD4^+^ T cells and monocytes. These blocks range from low availability of nucleotide pools for reverse transcription [45], translocation of the pre-integration complex to the nucleus [46], transcription of the provirus [47,48,49] and export of RNA [50]. While transcription of the provirus in resting CD4^+^ T cells and monocytes can be restricted by epigenetic modifications and chromatin structure [17,51], limiting levels of essential host factors for HIV-1 transcription, such as P-TEFb [52], also greatly contribute to the low permissivity of resting CD4^+^ T cells and monocytes to HIV-1 infection. Mechanisms that have been shown to regulate P-TEFb in CD4^+^ T cells and monocytes/macrophages are illustrated in Figure 1; the text below summarizes these mechanisms. 

### 4.1. Expression and Regulation of Cdk9

In contrast to Cdks involved in cell cycle regulation, Cdk9 activity and expression is generally not tied to the cell cycle, but is dependent on immune cell activation or differentiation. Thus, while the Cdk9 promoter has a high constitutive activity [53], Cdk9 protein expression was found to be induced in activated peripheral blood lymphocytes and differentiated macrophages [36], suggesting that Cdk9 expression is differentially regulated according to the demands of the cellular transcriptional program. In recent years, as discussed below, substantial evidence has been accumulated regarding the regulation of Cdk9. 

In addition to the originally identified 42-kDa protein, Cdk9 also has a minor 55-kDa isoform which arises from a transcriptional start site upstream of the 42-kDa Cdk9 coding sequence [54]. The two isoforms have different sub–cellular localization and expression patterns; for instance, immunofluorescence studies have shown that the 42-kDa Cdk9 is present throughout the nucleus with concentration in nuclear speckles [55,56], while the 55-kDa Cdk9 is more localized to the nucleolus [57]. In resting CD4+ T cells and monocytes, total Cdk9 is expressed at a relatively modest level and is upregulated upon cellular activation or differentiation [58]. However, when examining the individual isoforms, it is apparent that the expression of Cdk9 55-kDa generally does not change in response to the activation of primary lymphocytes, while levels of Cdk9 42-kDa are increased [57]; Cdk9 55-kDa is expressed at a very low level in monocytes and is induced upon macrophage differentiation, whereas Cdk9 42-kDa is present at high levels in monocytes. The functional activity of Cdk9 is also limited in resting cells, primarily by the use of post-translational modifications including phosphorylation of the Threonine 186 (Thr186) residue in the T-loop of Cdk9 [59] and other residues [60], ubiquitination [61], and acetylation [62].

The kinase activity of Cdk9 is heavily influenced by the phosphorylation status of the Thr186 residue in the conserved T-loop of the enzyme, as phosphorylation opens up the catalytic pocket of Cdk9, allowing for entry of ATP and the substrate [63,64,65], while dephosphorylation may regulate dissociation of the core P-TEFb complex with various binding partners [59]. While this is not the only Cdk9 residue to be phosphorylated, it certainly is critical for P-TEFb function, and it is likely that low levels of Thr186-phosphorylated Cdk9 may contribute to the refractoriness of resting CD4^+^ T cells and monocytes to HIV-1 infection and replication. Using an antiserum that specifically recognizes Cdk9 phosphorylated on Thr186 [66], we found that the level of Cdk9 T-loop phosphorylation was very low to absent in resting CD4^+^ T cells [67]. Using the same reagent, Wu and colleagues [68] reported that undifferentiated monocytes also had low Cdk9 T-loop phosphorylation. Interestingly, activation of resting CD4^+^ T cells rapidly and dramatically induced Cdk9 T-loop phosphorylation in the absence of new protein synthesis [67], while monocyte differentiation also appeared to increase levels of T-loop phosphorylation [68]. This implies that in resting cells and monocytes, Cdk9 T-loop phosphorylation is repressed either by the action of phosphatases, a yet unidentified repressor, or low concentrations of other co-factors [69]. For example, Cdk9 T-loop phosphorylation appears to be dependent on Ca^2+^signaling, with calcium/calmodulin-dependent kinase 1D (CAMK1D) acting upstream of the pathway [69]. SiRNA depletion of CaMK1D or small molecule inhibition of specific Ca^2+^ signaling pathway components reduced Cdk9 T-loop phosphorylation in primary CD4^+^ T cells [69]. In addition to Cdk9 Thr186, the phosphorylation status of Serine 175 (Ser175) has been reported to regulate Cdk9 kinase activity. Nekhai and colleagues provide evidence that in cell lines, Cdk9 Ser175 is phosphorylated *in vivo* and its dephosphorylation by PP1 activates HIV-1 gene expression [70]. 

Considering the pivotal role of P-TEFb in transcriptional elongation, it seems unlikely that a single protein or mechanism would be solely responsible for regulating Cdk9 T-loop phosphorylation. A case in point would be the role of phosphatases in regulating Cdk9 T-loop phosphorylation. We have identified two phosphatases, PPM1A and PPM1B, whose overexpression leads to increased levels of dephosphorylated Thr186 in the Cdk9 T-loop [66]. Immunoprecipitation experiments indicated that PPM1A associates with Cdk9 *in vivo* [66]. The 7SK snRNA ribonucleoprotein complex has been shown to sequester ~50% of phosphorylated Cdk9 in the cell [59], and *in vitro* phosphatase assays showed that while PPM1A could dephosphorylate Cdk9 without regard to P-TEFb’s association status with 7SK snRNA, PPM1B could only do so when 7SK snRNA was depleted [66]. Zhou and colleagues found that two other phosphatases, the PP1 catalytic subunit isoform PP1α and PP2B, cooperatively functioned to release P-TEFb from the 7SK snRNA complex by dephosphorylating the Cdk9 T-loop [71]. However, PP1α and PP2B-mediated dephosphorylation of the Cdk9 T-loop was examined in cells stressed by hexamethylene bisacetamide (HMBA) or UV treatment [71], unlike the dephosphorylation observed by PPM1A and PPM1B in actively growing cells [66]. Yet another phosphatase, PP1 can partially dephosphorylated Cdk9Thr186, although this phosphatase has high activity for the dephosphorylation Ser175—a positive modification of Cdk9 function [70] The stable expression of a protein inhibitor of PP1 (cdNIPP1) increased CDK9 phosphorylation on Thr186 and the association of CDK9 with 7SK RNA [72]. As phosphatases often have high substrate promiscuity, it is not surprising that multiple phosphatases can regulate Cdk9 T-loop phosphorylation, possibly in a manner dependent on cell type and growth conditions. Similar to the dephosphorylation of Cdk9 on Thr186, acetylation of Lys48 by GCN5 and P/CAF has also been shown to reduce P-TEFb kinase activity [62]. While these authors did not look at level of Thr186 phosphorylation in relation to acetylated Cdk9, immunoprecipitation experiments using cell lysates overexpressing Flag-HEXIM1 or Flag-Cyclin T1 did not show a difference in binding capacities between total and acetylated Cdk9 [62]. 

A structural study by Johnson and colleagues showed Cdk9 to be autophosphorylated in *cis* at Thr186 [60]. Purified Cdk9-Cyclin T1 was able to autophosphorylate Thr186 *in vitro*, while catalytically inactive and dominant-negative Cdk9 mutants failed to autophosphorylate, leading the authors to conclude that this process occurs in *cis* [60]. However, the autophosphorylation is relatively inefficient *in vitro*, raising the possibility that an activating kinase exists that can phosphorylate the Cdk9 T-loop. Indeed, the activity of such an activation kinase has been observed in nuclear extracts [59], but the identity of this elusive Cdk9 activating kinase remains to be determined. Additional work needs to be done to identify the functionally significant post-translational modifications of Cdk9, especially phosphorylation of Ser175.

HIV-1 infected persons who maintain undetectable viral loads in the absence of antiretroviral therapy are called elite controllers. CD4^+^ T cells isolated from this group of patients showed increased expression of the cyclin-dependent kinase inhibitor p21 relative to uninfected individuals and individuals with progressive HIV-1 infection. Knockdown of p21 in CD4^+^ T cells relieved a block in proviral transcription following infection of CD4^+^ T cells from elite controllers and resulted in increased kinase activity of Cdk9 on the RNAP II CTD Ser2 residues. P21 was also shown to co-immunoprecipitate with Cdk9, suggesting that p21 induction in elite controllers may contribute to blocking HIV-1 replication by inhibiting P-TEFb activity [74]. Interestingly, data from the Nekhai laboratory indicate that Cdk2 associates with Cdk9 [75] and inhibition of Cdk2 function by iron chelators reduces Cdk9 kinase activity [76]. Since p21 has been reported to inhibit Cdk2 activity [77], it is possible that a nexus of p21 and Cdk2 co-operatively regulate Cdk9 function. 

### 4.2. Expression and Regulation of Cyclin T1 and Cyclin T2

Cyclin T1 protein levels are low in resting CD4^+^ T cells but undergo a dramatic induction upon CD4^+^ T cell activation. Cyclin T1 protein expression is also dramatically increased upon monocyte to macrophage differentiation and is induced by HIV-1 infection in macrophages. Cyclin T1 levels in macrophages were observed to decline *in vitro* 1–2 weeks post-differentiation [78]; using proteasome inhibitors, it was found that this decrease in Cyclin T1 involved proteasome-mediated proteolysis [79]. The presence of a PEST sequence in a protein potentially marks it for rapid degradation via the proteasome pathway [80], and Cyclin T1 contains a PEST sequence at its carboxyl terminus and appears to be ubiquitinated in this and other regions of the protein [39,81].

The increase in Cyclin T1 protein levels following CD4^+^ T cell activation or macrophage differentiation appears to occur independently of an increase in CyclinT1 mRNA levels, suggesting a post-transcriptional regulation of CyclinT1 expression in resting T cells and monocytes. Indeed, we have found that Cyclin T1 is under microRNA (miRNA) regulation, with miR-27b, 29b, 150 and 223 decreasing Cyclin T1 in resting CD4^+^ T cells [82] and miR-198 repressing Cyclin T1 in monocytes [83]. In accordance with their repressive effects on Cyclin T1, these miRNAs are highly expressed in resting CD4^+^ T cells or monocytes when Cyclin T1 protein levels are low, but are down-regulated upon cell activation or differentiation, when Cyclin T1 protein dramatically increases. While functional binding sites within the Cyclin T1 3’UTR were identified for miR-27b and miR-198, the other miRNAs listed above may act indirectly to decrease Cyclin T1 protein expression, as we have been unable to identify direct binding sites for these miRNAs in the Cyclin T1 mRNA [82,83]; miR-29b, 150, and 223 have also been shown to bind to sites within HIV-1 RNA. All of the above miRNAs decrease HIV-1 replication when overexpressed [82,83,84,85], while inhibition of miR-150 and miR-223 have been reported to reactivate latent virus from resting CD4^+^ T cells isolated from aviremic patients [84]. Interestingly, a recent report by Matthews and colleagues identified a nuclear factor, NF90, which appears to be a positive regulator of Cyclin T1 protein expression [86]. NF90 binds to the Cyclin T1 3’UTR and stimulates translational initiation independently of the miRNA pathway [86]. NF90 has also been reported to be a TAR binding protein influencing HIV-1 Tat function [87]. These data suggest that the mechanism of Cyclin T1 post-transcriptional regulation is cell-type dependent, and is achieved via the coordinated action of miRNAs and other RNA binding proteins. 

Cyclin T2, the other cyclin partner of Cdk9 in P-TEFb, does not seem to be regulated to the extent of Cyclin T1. The expression of Cyclin T2 is relatively high and generally does not increase following activation of CD4^+^ T cells or macrophage differentiation [88]. Why, then, does the cell maintain two Cyclin partners for Cdk9? While it appears that these two Cyclins are largely functionally redundant, the expression of a subset of genes involved in cellular function and development appears to be specifically dependent on either Cyclin T1 or Cyclin T2. While Cyclin T1 and Cyclin T2 depletion using shRNAs does not affect cell viability [89,90,91], knockout of Cyclin T2 in mice is embryonic lethal and Cyclin T2 was shown to play a non-redundant role in murine embryonic stem cells [92]. We found very little redundancy between genes decreased by Cyclin T1 or Cyclin T2 knockdown in HeLa cells [89], and consistent with the fact that the majority of P-TEFb in cells contains Cyclin T1, we identified more genes responsive to Cyclin T1 [89,90,91] than Cyclin T2 depletion [89]. Cyclin T2 is expressed at high levels in adult human skeletal muscle cells and reportedly plays a role in muscle cell differentiation, especially of myocytes [93]. Cumulatively, these results suggest that the degree of redundancy in gene regulation by either Cyclin T1 or Cyclin T2 appears to be cell type-and species-dependent. 

In resting CD4^+^ T cells, Cyclin T1 and Cyclin T2 associate with Cdk9 even in the absence or at low levels of T-loop phosphorylation [67]. Cyclin T2 has two splice variants, T2a and T2b [94]. Thus, considering Cyclin T1 and the Cyclin T2 isoforms, both the 42 and 55-kDa isoforms of Cdk9, and the presence or absence of T-loop phosphorylation, altogether 12 core P-TEFb species have the potential to exist in cells. Six of these 12 P-TEFb species have kinase activity and can perform its transcript elongation function due to a phosphorylated Cdk9 T-loop, while HIV-1 transcription can only utilize two of these species, as Tat makes direct protein-protein contact with Cyclin T1 and not Cyclin T2 [40]. How HIV-1 evolved to utilize Cyclin T1 and not Cyclin T2 is not clearly understood, although it is likely that differences in their expression patterns and in the specific genes under their regulation might have influenced the development of this exclusivity [88,89,90,91,92]. 

## 5. The ‘Complex’ Diversity of P-TEFb

**Figure 2:**
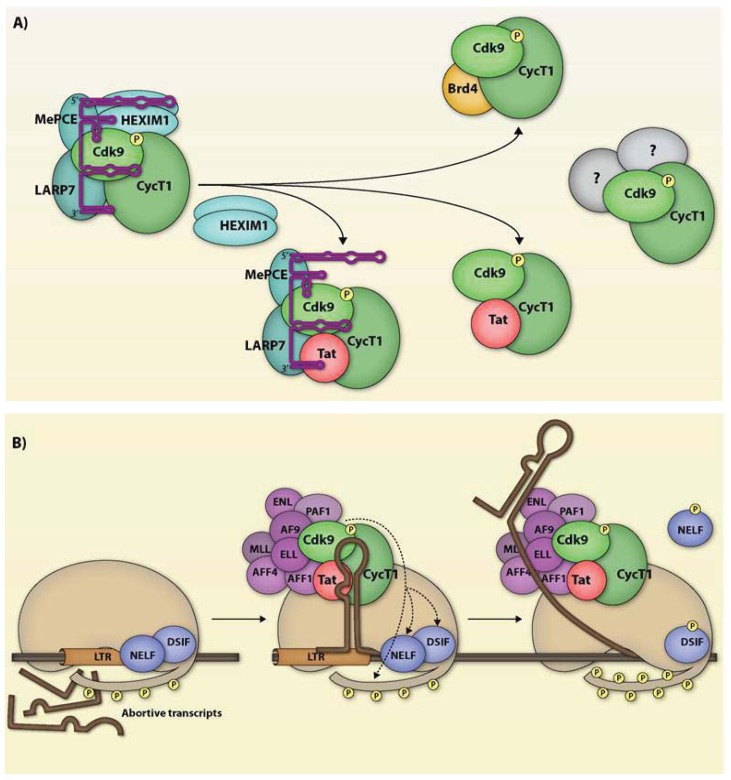
P-TEFb complexes. (**A**) Inactive P-TEFb is sequestered in the 7SK snRNP. Brd4 and Tat can both recruit P-TEFb directly from the inactive complex for cellular or HIV-1 transcription, respectively. Tat has also been found to associate with 7SK snRNP lacking the HEXIM dimers, hypothesized to be an intermediate of the P-TEFb recruitment process. Recently, P-TEFb has been shown to immunoprecipitate with a number of additional proteins, although the functional relevance of these associations has yet to be determined; (**B**) Prior to P-TEFb recruitment, proviral transcription proceeds inefficiently, resulting in the production of abortive transcripts. RNAP II processivity is highly increased following Tat-mediated recruitment of P-TEFb to the TAR RNA, where it also associates with the SEC. Cdk9 phosphorylates the negative elongation factors NELF and DSIF, resulting in the dissociation of NELF and the conversion of DSIF into a positive elongation factor, and the Ser2 residues of the RNAP II CTD, inducing efficient transcriptional elongation.

Productive infection of HIV-1 depends on the function of HIV-1 Tat and its recruitment of Cyclin T1-containing P-TEFb. The viral transactivator has to scavenge P-TEFb from within the cellular milieu since the core P-TEFb unit exists within multi-protein complexes. At least in HeLa cells, about 50% of P-TEFb is believed to be sequestered in the large 7SK snRNP complex [95,96,97,98] while most of the remaining P-TEFb associates with Brd4 [99,100]. The super elongation complex (SEC) is another multi-protein unit that has been shown to associate with P-TEFb and Tat [101,102]. Both Brd4 and Tat can recruit P-TEFb from 7SKsnRNP [103] recruiting it to cellular or viral promoters [100,104]. The P-TEFb that is recruited can enter into complex with other members of the SEC [19]. The role of these different P-TEFb complexes in HIV-1 gene regulation is illustrated in Figure 2.

### 5.1. P-TEFb and 7SK snRNP

In addition to P-TEFb, the 7SK snRNA complex contains HEXIM1/2 [59], LARP7 [105,106], MePCE [107] and a growing list of other proteins [108]. Within the 7SK snRNP, LARP7 inhibits the methyl transferase activity of MePCE, preventing the removal of the 7SK cap structure and thereby stabilizing the snRNA complex [107]. While phosphorylation of the Cdk9 T-loop appears to be a precondition for its association with the 7SK snRNP [59,65], the P-TEFb contained therein is functionally inactive, as Cdk9 is inhibited by binding to a HEXIM dimer [109]. In resting and activated CD4+ T cells, we have observed similar levels of HEXIM1 association with Cdk9 in immunoprecipitation experiments following a short time course of activation [67]. This association is significantly increased when resting CD4+ T cells are activated with prostratin for two days [88]. Thus this P-TEFb sequestration mechanism may have evolved to not only provide a readily deliverable source of active P-TEFb, but also to prevent promoter-proximally paused polymerases from inordinate activation.

### 5.2. P-TEFb and Brd4

The immune functions of CD4^+^ T cells and macrophages depend on their response to stimuli within an appropriate time frame. The direction of P-TEFb by Brd4 to cellular promoters [18] appears to be dependent on the Ser175 residue in Cdk9, as an alanine substitution was shown to abrogate Brd4 binding to P-TEFb [100]. Unlike the 7SK snRNA-bound P-TEFb, the Brd4-associated P-TEFb is transcriptionally active [99,100] although data from John Brady and colleagues suggest that Brd4 induced phosphorylation of CDK9 at Thr29 residue results in a transient inhibition of P-TEFb activity and its subsequent dephosphorylation reverses this inhibition [110]. In an elegant study using Toll-like receptor (TLR)-inducible gene expression in macrophages, Medzhitov and colleagues [4] showed that a group of cellular genes classified as primary response genes (PRGs) recruit P-TEFb via Brd4 within an hour of lipopolysacchride (LPS) stimulation. Most PRG promoters exhibited RNAP II occupancy with phosphorylated Ser5 even in the absence of stimulation. Secondary response genes (SRGs), which require *de novo* protein synthesis and epigenetic remodeling at their promoters for their expression [111], showed a more delayed response to LPS than PRGs and lacked RNAP II promoter occupancy prior to stimulation. The permissive features of PRG promoters may be cell type-independent since they are shared between macrophages, MEFs, and embryonic stem (ES) cells [9]. Using ChIP-chip, Guenther and colleagues [9] found that while many genes in ES cells undergo transcriptional initiation, two histone modifications associated with actively transcribed genes, H3K36me3 and H3K79me2, were detected almost exclusively downstream of promoters that generate detectable full-length transcripts. Many inactive genes showed a low but significant level of RNAP II binding at their promoters, and a similar pattern was detected in primary human hepatocytes and B lymphocytes. Thus, data from these two studies [4,9] suggest that RNAP II is poised on many inactive genes, particularly those that are rapidly responsive, and simply awaits the right transcriptional program signals to begin making full length transcripts.

### 5.3. P-TEFb and the SEC

Identification of the components of the SEC using a sequential affinity purification scheme included the core P-TEFb, MLL, AF9, AFF4, AFF1, ENL, ELL and PAF1 [101,102]. Chromatin immunoprecipitation (ChIP) experiments showed the association of P-TEFb, AF9, ELL and PAF1 with Tat [102]. Depletion of AF9 reduced Cdk9 catalytic activity in the SEC, while the depletion of Cdk9 reduced the amount of ELL and AFF1, suggesting that Cdk9 is involved in SEC-Tat assembly and P-TEFb function in this complex is dependent on AF9 [102]. Phosphorylation of AFF1, AFF4, AF9 and ENL seems to be a requirement for the assembly of the SEC [102], and it remains to be seen whether these SEC component proteins are Cdk9 substrates.

### 5.4. P-TEFb and Other Multi-Protein Complexes

Multi-protein complexes carry out most cellular processes [112]. In addition to the complexes reviewed above, a recent high-throughput IP-mass spectrometry (HT-IP/MS) analysis identified a number of additional CycT1/Cdk9 complexes whose function remains to be elucidated [113]. The P-TEFb-associated complexes in the IP-mass spectrometry study are involved in RNA splicing, transcription termination, and nuclear pores, suggesting that P-TEFb may also play functional roles in these processes or at these cellular locations [113]. The functional significance of the association of core P-TEFb into the complexes identified in the HT-IP/MS remains to be elucidated.

## 6. P-TEFb and HIV-1 Latency

Viral latency is defined as the reversible non-productive infection of host cells. In the case of HIV-1, viral latency is primarily seen in long-lived resting memory CD4^+^ T cells, although certain other cell types can also harbor latent virus. The robust replication of HIV-1 following infection of activated CD4^+^ T cells and the accompanying immune response is cytopathic [114]. However, some infected CD4^+^ T cells may survive long enough to revert back to a resting state, thereby establishing viral latency. Clinically, it is likely that the virus may reside in sites which offer barriers to antiviral drug access [115]. A hallmark of HIV-1 latency is the low or nearly absent viral gene expression, indicating that latency is primarily maintained at the level of transcription. Latent provirus has mostly been detected in actively transcribed genes [116,117,118] implying post-integration transcriptional repression. Given the importance of Tat in viral transcription, it has been suggested that low levels of Tat are a primary driver of the establishment and maintenance of latency, while re-activation is also driven by increases in Tat expression. Expressing Tat *in trans* prevents the establishment of latency in a Jurkat cell model [119], while stochastic fluctuations in the positive Tat feedback loop can decrease Tat expression and drive viral entry into latency [120,121]. Assaying the Tat proteins derived from CD4^+^ T cells taken before and after the initiation of HAART showed post-HAART enrichment for Tat with impaired transactivation ability [122]. As the Tat isolated after patients had achieved undetectable viral loads presumably derives from latently infected cells, this suggests that a partially defective Tat can push cells into latency during *in vivo* infection. This further suggests that P-TEFb, the cellular mediator of Tat function, may also contribute to viral latency. Treatment of cells with hexamethylbisacetamide (HMBA), an activator of P-TEFb, can induce latent proviruses [123,124,125], and T-cell receptor-mediated induction of P-TEFb has been found to be essential for the activation of latently infected primary CD4^+^ T cells [126]. Integration of HIV-1 into the host genome renders proviral transcription susceptible to epigenetic modifications. DNA and histone methylation [127,128] and the deacetylated histones [129] status of the host genome can influence HIV-1 transcription. We direct readers to an excellent review by Mbonye and Karn for a detailed discussion of epigenetic control of HIV-1 latency [130].

A technical limitation of primary cells for *in vitro* HIV-1 latency experiments is the inability of the culture system to support long-term CD4^+^ T cells survival. In recent years, however, a number of *in vitro* primary cell systems have been developed that circumvent this technical limitation and allow mechanistic studies into how HIV-1 latency is established and maintained, as well as how latent proviruses can be reactivated. The feeder cell-line, H80, has been used to maintain viability of primary activated CD4^+^ T cells for up to more than 5 months [131]. The system devised by Lassen *et al.* allows for a rapid generation of latently infected CD4^+^ T cells [132]. The model developed by Saleh *et al.* allows for resting CD4^+^ T cells to be efficiently infected following incubation with one of the CCR7 ligands, CCL19 or CCL21 [133]. Siliciano’s group used the lentiviral transduction mediated stable expression of the anti-apoptotic factor, Bcl-2, to maintain cell viability *in vitro* [134]. These cells could then be activated, infected with HIV-1 and allowed to return to resting state while retaining viability [134]. The Planelles laboratory employed cytokines to drive naïve CD4^+^ T cells to non-polarized T helper 1 or T helper2 phenotypes, allowing studies of HIV-1 latency in a physiologically relevant memory T cell type [135]. 

In addition to these recently developed primary cell systems for studies of viral latency, the Jurkat CD4+ T cell line provides additional *in vitro* models for mechanistic studies. Jurkat cell line latency models developed by the Karn [119] and Verdin laboratories [136] have proved useful for mechanistic and biochemical experiments with host co-factors to understand the intricacies of viral latency and reactivation. In both these models, clonal cell lines are derived following infection with HIV-1 based retroviral vector. The vectors also encode for GFP allowing for easy monitoring of proviral expression by flow cytometry. In the absence of stimulation, these cell lines show almost no GFP expression. Treatment with TNF-α or other activation agents induces GFP expression that dissipates with time and can be reactivated by further treatment with TNF-α or other activators. 

In conclusion, HIV-1 transcription is highly dependent on factors like P-TEFb and NF-κB whose activity is low or repressed in resting cells. When these infected resting cells respond to antigens by initiating the activation program, the HIV-1 provirus can be efficiently transcribed by using induced cellular proteins like P-TEFb. The low activity of P-TEFb in HIV-1 latently infected cells could also play a role in the maintenance of viral latency. Cyclin T1 expression is low in resting CD4^+^T cells, in part due to the action of miRNAs like miR-27b [82], and in monocytes, where high expression of miR-198 correlates with low Cyclin T1 levels [83]. Cdk9 T-loop phosphorylation appears to be repressed in resting CD4^+^ T cells and monocytes [67,68,69]. The effects of phosphatases like PPM1A [66] and PP1α [71] and the yet to be identified Cdk9 activating kinase on the maintenance of latent HIV-1 reservoirs are still to be determined. CD34^+^ bone marrow cells have been shown to harbor latent provirus, although the infection efficiency of these cells is very low [137]. However, a recent study using real time PCR and HIV-1 co-culture assays did not find viral DNA in highly purified CD34^+^ progenitor cells from bone marrow aspirates of HIV-1 patients on antiretroviral therapy [138]. It is possible that P-TEFb restriction in addition to other factors such as the chromatin epigenetics in these cells and other reservoirs could be contributing to the establishment of HIV-1 latency. 

## Avenues for Future Research

1. How many miRNAs individually and cooperatively regulate Cyclin T1 in CD4^+^ T cells and macrophages? What is their range of targets and how do these targets play into P-TEFb function?
2. Does HIV-1 Tat utilize the novel Cdk9/Cyclin T1 complexes identified by HT-IP/MS?
3. How can P-TEFb expression and function be manipulated (e.g., by inducing Cdk9 T-loop phosphorylation or using antagomiRs to inhibit Cyclin T1 responsive miRNAs) to reactivate latent provirus?
